# Evaluation of public submissions to the USDA for labeling of cell-cultured meat in the United States

**DOI:** 10.3389/fnut.2023.1197111

**Published:** 2023-09-08

**Authors:** Morgan Failla, Helene Hopfer, Josephine Wee

**Affiliations:** Department of Food Science, The Pennsylvania State University, University Park, PA, United States

**Keywords:** cellular agriculture, food policy, labeling, meat, public comment analysis, text analysis

## Abstract

With the rapid advancement of cell-cultured meat processing technologies and regulations, commercialization of cell-cultured meat to market shelves requires the implementation of labeling that informs and protects consumers while ensuring economic competitiveness. In November 2022, the United States Food and Drug Administration (FDA) completed its first pre-market consultation of cell-cultured meat and did not question the safety of these products for human consumption. As of June 2023, commercialization of cell-cultured meat products has become a reality in the United States. To derive potential label terms and gain insight into how different stakeholders refer to these novel products, we analyzed 1,151 comments submitted to the 2021 U.S. Department of Agriculture’s Food Safety and Inspection Services (USDA-FSIS) call on the labeling of cell-cultured meat and poultry. Our first aim was to systematically assess the nature of comments with regards to their length, cited references, and supplemental materials. In addition, we aimed to identify the most used terms to refer to these products through text analysis. We also asked how these analyses would vary by affiliation category and economic interest. Using the listed organizations for each comment, we first determined financial ties: 77 (7%) comments came from those with an economic interest, 12 (1%) of the comments did not have an identifiable economic interest, while for the remaining 1,062 (92%) comments economic interest could not be determined. We then grouped comments into affiliation categories. Cell-cultured meat companies and animal welfare non-profits had the highest median word count, whereas comments from the unknown affiliation category had the lowest. We found across all comments the predominantly mentioned potential label terms, in descending order, to be *cultured meat, lab-grown meat, cultivated meat, cell-cultured meat, clean meat, and cell-based meat*. While all label terms were discussed throughout overall submissions, percentages of comments mentioning each term differed between affiliation categories. Our findings suggest differences in how affiliation categories are discussing cell-cultured meat products for the US market. As a next step, the perception and acceptance of these terms must be evaluated to identify the optimal label term regarding the information and protection provided to consumers while ensuring economic competitiveness.

## 1. Introduction

Alternative meat innovation has advanced with increased awareness of the traditional meat farming industry’s impact on global sustainability and food security. US Americans are among the top five consumers of beef, veal, pork, and poultry meat (1). The impact of traditional meat production on greenhouse gas (GHG) emissions and water use poses global sustainability issues (2). Beef farming in particular represents almost half of GHG emission associated with agriculture in the United States (3). While research has prioritized decreasing environmental impacts of traditional meat production, several studies report that a majority of consumers are unaware of negative implications of meat production and consumption (4–6). Currently, few consumers choose meat alternatives to replace traditionally farmed meat due to their dissimilarity in terms of flavor and texture (7). Food insecurity has increased globally since the COVID-19 pandemic (8). The rising world population also imparts further issues toward adequate meat production; global food production must increase by 70% by 2050 to meet demands (9). The disruption of the economy from the pandemic paired with the influx of the world population creates an obstacle for consumers to purchase and consume protein-rich foods with a lower environmental impact (10).

Cell-cultured meat^1^ has emerged as an alternative and parallel production to traditional meat due to the potential positive impact on global sustainability, paired with a sensory experience that mimics that of traditionally farmed meat (11). However, it remains unclear how able the cell-cultured meat industry will be to successfully manufacture products that at the same time meet these goals. (12). As production processes are still being optimized, little is known of the taste, nutrition, safety, and environmental impact of large-scale production of these products (13, 14). Further, differences in acceptance of cell-cultured meat hinders broad public appeal (15, 16). Cell-cultured meat is produced through a process starting by (1) identifying and isolating target cell and tissue, (2) selecting and expressing target cells, (3) culturing of cells, (4) collecting of cell biomass, and (5) processing into a meat product (17). While this process has the potential to create meat products while using less water, consumer perception varies, which may be attributed to differences in consumer attitudes including food technology neophobia (13).

In the United States, the FDA approved cell-cultured meat production in November 2022 followed by the USDA’s approval for commercialization in June 2023 (17, 18). While the regulatory barriers for production and sales of cell-cultured meats have been lowered, there is a gap in determining how these products should be labeled. In 2021, the United States Department of Agriculture’s Food Safety and Inspection Services (USDA-FSIS) published an advanced notice of proposed rulemaking (ANPR), open to public submission. They sought public input regarding the labeling of cell-cultured meat and poultry (19). The purpose of the ANPR is to establish the intent of a new rule or regulation and allow for public comment *via* an open response forum to gain insights into public opinion to facilitate rule development. There are no requirements regarding who is able to submit a comment. According to the Federal Register, an “*Advance Notice is a formal invitation to participate in shaping the proposed rule and starts the notice-and-comment process in motion*” (20).

Specifically, the ANPR gathered public comments on cell-cultured meat with 14 questions provided by FSIS (see Supplementary Table S1). These questions prompted responses on potential labeling strategies, the impact of the label terms on the meat market, and how these labels would differentiate cell-cultured meat products from traditionally farmed meats (19). To understand the views of the public, we analyzed ANPR comments with text analysis tools to gain insight into how proposed labels differ between affiliation categories of comment submitters.

Regulation and identification of cell-cultured meat within the standards of identity of meat must be implemented for consumers to make informed purchase decisions. There are two sections within the Code of Federal Regulations in which cell-cultured meat must be addressed, namely, how meat is defined and how meat is labeled. The Federal Meat Inspection Act, found in 9 CFR301.2, legally defines foods including meat, meat byproduct, and meat food product (21). While cell-cultured meat does not abide by the definitions for meat or meat byproduct as it is not “part of the muscle of cattle” or “any part capable of use as human food” (21), it certainly would fit into the definition of a meat food product, “any article capable of use as human food which is made wholly or in part from any meat or other portion of the carcass…” (21). This legal ambiguity of what differentiates traditional meat from cell-cultured meats is part of the challenge of how to label these products, not only in the United States but around the world (22).

Labeling definitions and characteristics of meat, meat byproducts, and meat food products are regulated as described in 9 CFR 317.2 (23). There it is stated that for any product with no common or usual name, a descriptive designation used as a product name must clearly and completely identify the product. Further, meat preparation processes (e.g., smoking, salting) must be identified on the label and industry relevant terms (e.g., “picnic,” “cala”) shall not be used as the product name unless paired with descriptive terms (e.g., flavorings, marinades), or with a list of ingredients, to ensure transparency (23). With cell-cultured meat companies, such as Upside Foods and Good Meat, recently receiving regulatory clearance for production and sales, stakeholders must consider these parameters when establishing labeling strategies. Such strategies could include omission of the word ‘meat’ all together and/or use of another term, such as ‘protein’.

With commercialization of cell-cultured meat in the United States, meat industry stakeholders vary in their support for these new products. Due to the potential for cell-cultured meat to take over a percentage of the market share within the traditional meat industry, established traditional meat farmers may experience negative impacts in terms of food security and profits (24). On the other hand, success in the cell-cultured meat industry could be dependent on cell lines obtained from healthy cattle raised by traditional meat farmers, therefore transparency and communication strategies must be enforced to ensure fairness and equity between these two groups. Here, we seek to identify differences in proposed labeling strategies submitted to the ANPR as a function of meat industry stakeholder groups and affiliations (e.g., cell-cultured meat vs. traditional meat companies).

An interesting case in this effort represents Tyson Foods, a top investor in cell-cultured meat as well as an established leader in the traditional meat farming industry. Tyson has invested in companies developing cell-cultured meat such as Upside Foods, Memphis Meats, and Future Meat Technologies (25–27). As such, Tyson Foods’ comments on how cell-cultured meat should be commercialized and labeled presents an opportunity to analyze how potential opposing viewpoints could be resolved. For these reasons, we chose to analyze Tyson Foods’ submission separately.

We outlined three main objectives for this study. First, we aimed to determine the extent and disclosure of comments as they relate to economic interests and affiliation. We hypothesized that those with a stated economic interest will have a higher percentage of submissions compared to those without identified financial ties. Here, we further defined an economic interest as those who experience impact to their economic status with the commercialization of cell-cultured meat. Further, we also hypothesized that different affiliate categories will differ in their submissions with regards to length and extent of providing scientific evidence and supplemental materials, with cell-cultured meat companies hypothesized to provide more external reference citations than traditional meat companies.

For the second objective, to identify the predominant labeling terms used for cell-cultured meat across the different affiliation categories, we hypothesized that the cell-cultured meat industry and traditional meat farming industry will refer to these novel products in their submission using different label terms. Further, we evaluated the diversity of label term constructs (i.e., hyphenated, preceding, and root terms). For example, label term constructs could be “cell-cultured meat,” “man-made protein,” which were similarly analyzed to create a comprehensive list.

Last, the submission received by Tyson Foods was treated as a special case due to the economic interests of this company in both traditional meat farming and cell-cultured meats. Analyzing this submission as described above, we hypothesize that the Tyson Foods submission will mention label terms similar to the predominant labels mentioned by the traditional meat farming affiliation group due to their longer history in the traditional meat industry.

Outcomes from a systematic and scientific evaluation of the comments submitted to the USDA-FSIS ANPR will provide an overview of submission extent, disclosure, and label term use for industry and regulatory professionals to gain insight on current terminology regarding cell-cultured meat in various sectors. Results from our systematic analysis could form the basis of future consumer insights research, to assess perception and acceptance of different labels for these novel food products. Our analytical method, pairing automated text analysis tools with manual evaluation, allows for a comprehensive assessment of terms used to discuss these products.

## 2. Materials and methods

### 2.1. Dataset

On 3rd September 2021, the USDA-FSIS started collecting open responses *via* an ANPR regarding “labeling of meat or poultry products comprised of or containing cultured animal cells.” The proposed rule contained 14 items (see Supplementary Table S1) for public comments including how products should be labeled, how label terms impact consumer choice, standards of identity, and the meat market. The submission period was initially opened until 2nd November 2021, but extended until 2nd December 2021. A total of 1,207 comments were received during this timeline, of which 1,180 were available for download. The remaining 27 comments were inaccessible due to the USDA-FSIS quality standards; no further information was provided. The data was accessed for analysis on 12th September 2022 *via* the USDA-FSIS website and exported to Microsoft Excel. While most comments were viewable in the Excel file, some comments were extracted by downloading pdf and/or Microsoft Word files that were available in the Excel file as downloadable attachments. All data analyzed in this study were retrieved from Regulations.gov, the United States Federal government website document repositor.^2^

### 2.2. Data analysis

A comprehensive outline of our data analysis procedures is summarized in Supplementary Figure S1. Upon manual inspection of all submissions, those that were duplicated (*n* = 18), unrelated (*n* = 2), blank response (*n* = 2), or requesting an extension for the submission period (*n* = 5) were omitted from further analysis. One submitted Excel file, containing 6,028 identical entries, was also omitted from analysis. Supplemental submission materials were not analyzed as part of the median word count analysis. In addition, citations, welcoming introductions, repeated USDA-FSIS proposed questions, and closing remarks were manually removed to standardize submission comments to only contain information regarding labeling of cell-cultured meat. After cleaning, 1,152 comments, including the Tyson Foods submission, were used for analysis. Over 99% of submissions (1,143 out of 1,152) were made by individuals or entities residing in the United States, based on the stated location for each comment.

Several inferences were made for our analysis. Economic interest of submissions was evaluated to determine the extent and disclosure of comments relative to their economic relation to the cell-cultured meat industry. Economic interest was determined by manual web and/or social media searches and established using one or more of the following parameters:

1. Direct connections to the cell-cultured meat industry, e.g., cell-cultured meat companies.
2. Cell-cultured meat production and sales may directly affect their market share in the meat industry, e.g., traditional meat farmers.
3. Economic interest was outwardly stated in comment.
4. Non-profit organizations who represent the interests of companies satisfying points 1 or 2, due to dependency on each other for economic success.
5. Research organizations who receive funding related to the cell-cultured meat industry.
6. Investments in companies satisfying 1 or 2.

Affiliation categorization was determined by manually researching each organization and sorting submissions into affiliation categories that were identified *via* discussions by the author team (see Supplementary Table S2): cell-cultured meat companies, traditional meat farmers, research organizations, farmer advocacy groups, federal, state, and government agencies, animal welfare non-profits, other, and no affiliation. Cell-cultured meat companies included companies that produce cell-cultured meat. Traditional meat farmers included companies that produce traditionally farmed meat. Research organizations included organizations that perform research related to cell-cultured meat, agricultural sustainability, human nutrition, food safety, and genetically modified foods. Farmer advocacy groups included organizations that advocate for traditional meat farming practices. Federal, state, and government agencies included responses from those agencies and representatives of those agencies. Animal welfare non-profits included organizations that advocate for ethical farming practices. Other was recorded when there was an affiliation listed but it did not fit into the previously listed categories. Unknown affiliation included those who commented that did not identify an affiliation. This included 131 submissions made from differing individual submitters that started “As an Arkansas farmer …” Due to lack of disclosure of an organization, these submissions were categorized in the ‘unknown’ category.

Once submissions were cleaned and categorized, a combination of manual and automated analyses in R, NVivo, and Microsoft Excel was conducted across all comment submissions and per affiliation category. The number of scientific references and supplemental information was manually quantified and recorded for each submission. This was performed by examining footnotes, bibliographies, and in-text citations. The percent reported was calculated by counting the number of comments containing cited references for each affiliation category. Mean citation count was quantified by reporting the total number of references for each submission and calculating the mean for each category. Supplemental information included slide presentations, attached research articles, and summary booklets. Supplemental information counts were quantified from manually counting and calculating total submissions that attached supplemental information counts. Analysis was performed in R (V.4.2.1), using the RStudio environment (V.2022.7.2.576, Boston, MA) with the additional packages ggplot (28), dplyr (29), tidytext (30), and ggpubr (31). We used dplyr and tidytext to evaluate the word count for each group by affiliation and economic interest reported as median and range word count. Comments were analyzed directly in RStudio, as they were within the cell character limit of Excel (32,767 characters). However, two comments (Good Food Institute, Harvard Animal Law & Policy Clinic/Harvard Food Law and Policy Clinic), which had content larger than this were analyzed manually *via* Microsoft Word.

Then each submission was analyzed for commonly mentioned label terms (e.g., cell-cultured, cultivated, clean, etc.) – this was done in step-wise manner using NVivo (version 12, QSR International, Burlington, MA): First, common words and phrases were identified surrounding the most common word mentioned ‘meat’. We used the ‘word tree’ function to identify the hyphenated and preceding terms to ‘meat’. Then each term was evaluated by ‘text search queries’ to identify the most common label term constructs used overall. We further performed a forwards and backwards analysis of preceding and hyphenated terms by word trees to evaluate root terms used to discuss these products. An overview of label term constructs was recorded in Excel (see Supplementary Table S3).

Using the most common label terms identified in overall comments, the Microsoft Excel advanced search was used to obtain counts of comments mentioning each term. All text search analysis was performed by searching for the full label term construct. The results were recorded in Excel for data visualization in R. We analyzed the percentage of submissions mentioning each label term overall and by affiliation category. To compare between affiliation categories, which differed in number of submissions, we report percentages of submissions within each affiliation group mentioning each label term. This was calculated by quantifying the number of submissions that mentioned each label within each affiliation category, divided by the total submission count for each category, and multiplied by 100. The same analysis was completed for the Tyson Foods submission. However, for Tyson Foods we did not calculate a percentage since *n* = 1 in this case.

## 3. Results

### 3.1. Economic ties and affiliation do not predict total submission count or word count

Our first research question addressed the extent and disclosure of comments related to economic interest. Extent is defined as the length and content of the submission which we evaluated using word count, number of references, and supplemental information. When there was an organizational tie which could identify economic interest, we used the term ‘disclosure’ to identify this group. We defined economic interest as those who have a financial tie to the cell-cultured meat or traditional meat farming industry (see Materials & Methods section). We hypothesized that those with economic interest will have a higher percentage of submissions both in number as well as length (i.e., median word count) in comparison to those without an identified economic interest. However, our analysis shows (Supplementary Table S4) that only 77 (6.7%) comments were submitted by an entity with an identifiable economic interest, 12 (1.0%) comments were entered by those without an identified economic interest, and the vast majority, 1,062 comments (92.3%) were submitted where the economic interest could not be identified (e.g., anonymous submission or submission as a private citizen).

Table 1 provides an overview of the origin and characterization of submissions by affiliation category. The parameters of comment composition (e.g., word count, number of references, and extent of supplemental information) was hypothesized to predict economic interest. We found that affiliation category did not impact submission extent or disclosure. Percentages of submissions by affiliation were calculated for each category and reported in Table 1. The majority of comments came from those with unknown affiliation (n = 1,062; 92.3% of submissions) which also had the lowest median word count of 68, or roughly one paragraph (Table 1). Cell-cultured meat companies made up 1.2% of submissions and had the second largest median word count of 2048, or roughly 2.5 letter sized pages single spaced (Table 1). The lowest number of submissions came from traditional meat farmers with 2 submissions (0.2%), neither of which included references or supplemental information, and had a median word count of 1,208. The greatest number of comments from those with a listed affiliation came from farmer advocacy groups, who submitted 37 comments (3.2%), with a median word count of 756 (Table 1). All other affiliations had comparable submission numbers ranging from 2 to 14 submissions (Table 1). The highest median word count of 2,379 were made by animal welfare non-profits who also had the highest mean citation count (Table 1).

**Table 1:** Presence of affiliations, citation of scientific evidence, supplemental information, and median word count.

Affiliation category	% of Total (*n*)	% Citing references (*n*)	Mean citation count	Median word count (Range)	Comments with supplemental information
Cell-cultured meat companies	1.2 (14)	64.3 (9)	8.1	2048 (283–4,614)	2
Traditional meat farmers	0.2 (2)	0 (0)	0	1,208 (848–1,567)	0
Research organizations	1.0 (11)	90.9 (10)	7.8	1,337 (359–5,990)	1
Farmer advocacy groups	3.2 (37)	13.5 (5)	1.1	756 (202–4,771)	1
Animal welfare non-profits	0.3 (4)	75.0 (3)	18.8	2,379 (626–3,291)	1
Government agencies	1.0 (11)	9.1 (1)	0.8	642 (323–1969)	0
Other	0.9 (10)	30 (3)	15.5	742 (66–8,731)	1
Unknown affiliation	92.3 (1062)	0.5 (5)	0.1	68 (1–4,609)	6

Percent citing references and mean citation counts differed greatly both by economic interest and affiliation: we found that those without identified economic interests (*N* = 12) had the highest mean citation count (M = 19.2), highest percent citing references (32%), and highest median word count (Mdn = 1766) (Table 1). The affiliation category with the highest percent of cited references included research organizations – of the 11 submissions in this group 90% cited references in their comments. The inclusion of supplemental material, as previously defined as additionally submitted materials together with the comment, did not differ by economic interest or affiliation categories (Supplementary Tables S1, S3). However, the comments for which no affiliation could be assigned showed the highest number of comments with supplemental information (Table 1). Upon further manual inspection these included PowerPoint presentations, figures, and peer-reviewed scientific manuscripts.

### 3.2. The same top 6 label terms were identified across all comments, however, label term mentions differ greatly across affiliation categories

**Figure 1:**
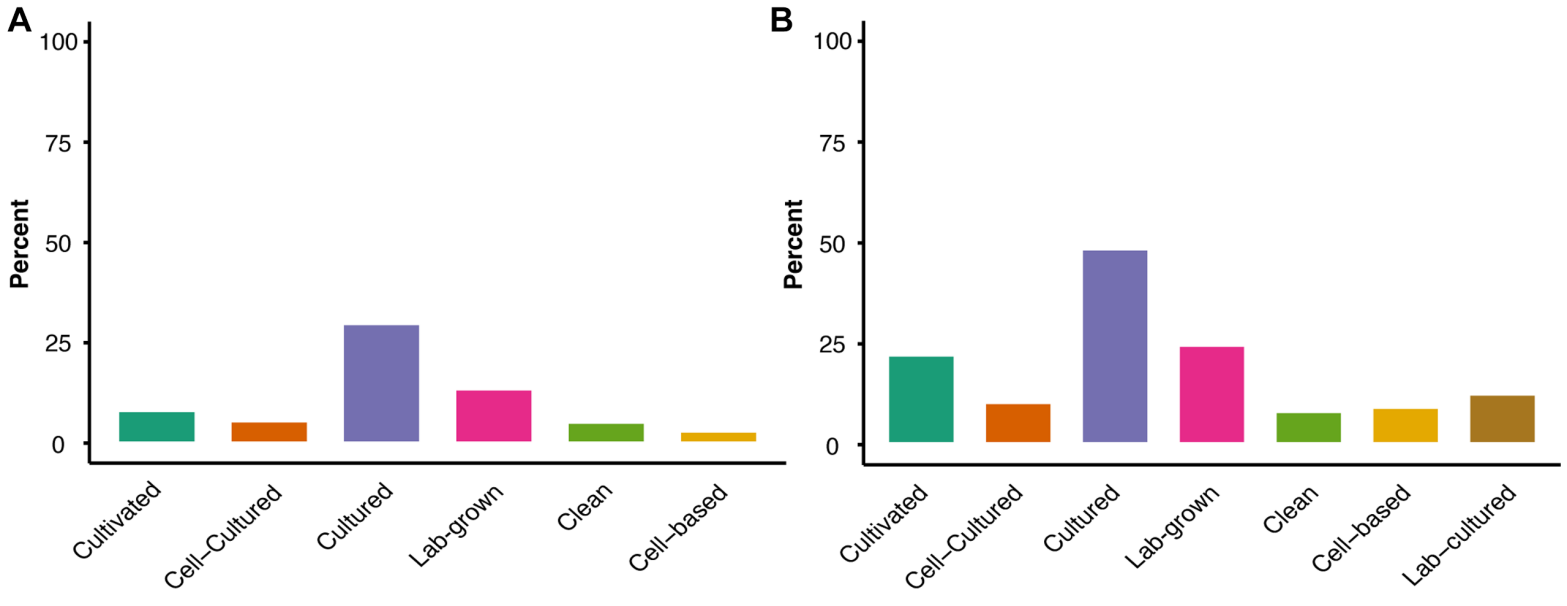
Overall percentages of submissions mentioning top label terms. **(A)** Overall percent of comments mentioning each hyphenated and/or preceding label term together with the root term ‘meat’. **(B)** Overall percent of comments mentioning each hyphenated and/or preceding label term, analyzed without the root term ‘meat’.

Our second research question assessed how different commenters referred to these novel food products in their submissions, as an indirect way of identifying potential labels for these products. Using NVivo to identify the top label terms, we found the most widely used overall, in descending order of percent of comments mentioned, to be *cultured meat* (29.8%), *lab-grown meat* (13.6%), *cultivated meat* (8.2%), *cell-cultured meat* (5.6%), *clean meat* (5.2%), and *cell-based meat* (3%) (Figure 1A). These top 6 terms accounted for 2/3 of all comments.

Due to the ambiguity in application of the current standards of identity for meat, potential labels could also omit the root term ‘meat’. Therefore, an additional analysis was carried out to identify root terms other than ‘meat’ or no root term at all, across all submissions and by affiliation category. Upon analyzing the top 6 hyphenated and preceding terms regardless of root term (i.e., searching without the root term ‘meat’), a similar rank order was found, except for *cell-based* and *clean*. Compared to analysis including the ‘meat’ root term, mentions of *cell-based* and *clean* switched rank order, with *cell-based* (9%) having higher mentions than *clean* (8%) (Figure 1B). The analysis without the root term *meat* also identified the label *lab-cultured* as a top label term (12%) (Figure 1B), as it was referenced by the unknown and government agencies affiliation groups in conjunction with the root term ‘protein’.

**Figure 2:**
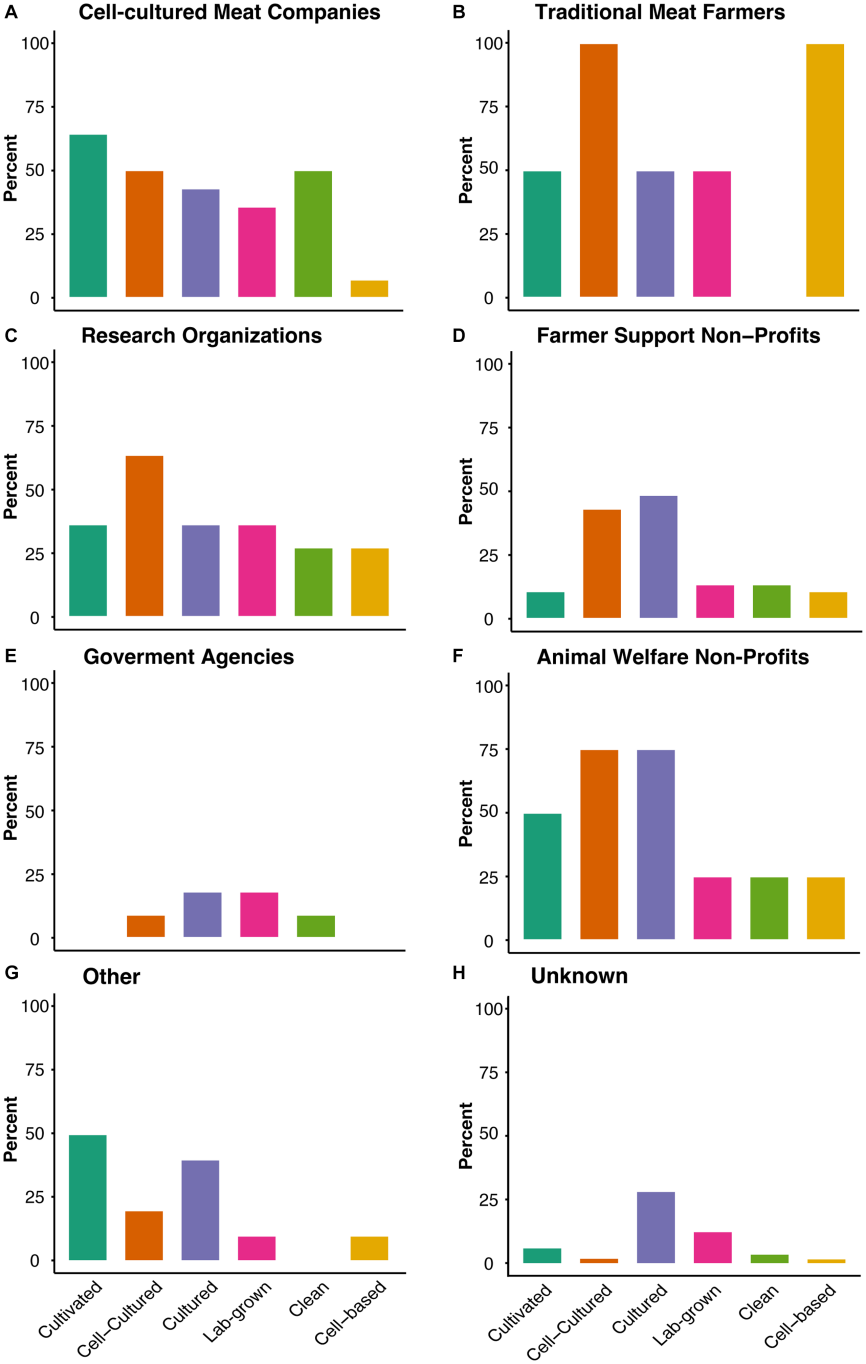
Percentages of submissions mentioning top label terms by affiliation. The percentages of submissions mentioning each of the top label terms in conjunction with the root term ‘meat’ was calculated for **(A)** cell-cultured meat companies (*n* = 14), **(B)** traditional meat farmers (*n* = 2), **(C)** research organizations (*n* = 11), **(D)** farmer advocacy groups (*n* = 37), **(E)** animal welfare non-profits (*n* = 4), **(F)** government agencies (*n* = 11), **(G)** other (*n* = 10), and **(H)** unknown affiliations (*n* = 1,062).

Using the top 6 terms in conjunction with the root term ‘meat’ identified in our analysis, we then focused on our second research question of how percentages of label terms mentioned in comments might differ across affiliation categories (Figure 2). To better understand the use of the hyphenated and preceding label terms, we further analyzed them without the root term ‘meat’ in Supplementary Figure S2. It was observed that overall trends of label mentions for each affiliation category were similar when analyzed with and without the root term ‘meat’ (Figure 2, Supplementary Figure S2). Among the mentions of the top 6 terms, differences in percentages are apparent between affiliation categories: While cell-cultured meat companies predominantly used the term *cultivated meat* (64.3%), all other terms were mentioned between 25–50%, except *cell-based meat* which was mentioned in less than 10% of submissions (Figure 2A). On the other hand, traditional meat farmers primarily used the terms *cell-cultured meat* and *cell-based meat* 100% of the time (Figure 2B). However, it is important to note that only 2 submissions made up the traditional meat farmers affiliation category. Interestingly, research organizations mentioned *cell-cultured meat* in 63.6% of comments (Figure 2C). Farmer support non-profits, the affiliation category with the highest number of submission (*n* = 37), mentioned the terms *cell-cultured meat* and *cultured meat* in a moderate percent of submissions (43.2, 48.6% respectively) (Figure 2D). Similarly, *cultured meat* and *cell-cultured meat* were the most frequently (both 75%) mentioned terms for submissions made by animal welfare non-profits (Figure 2F). Those categorized as “other” moderately mentioned *cultivated meat* (50%) and *cultured meat* (40%) (Figure 2G), while those with an unknown affiliation mentioned all terms sparingly (less than 25%), with *cultured meat* being the most commonly used term (28.7%) (Figure 2H). The comments within the unknown affiliation category beginning with “As an Arkansas farmer…” all proposed the term *lab-cultured protein*. Therefore, they did not affect the results in Figure 2, but contributed to the findings of Figure 1B and Supplementary Figure S2 indicating *lab-cultured* as a top hyphenated and preceding label term when analyzed without the root term ‘meat’.

Only 3 of the 6 label terms were mentioned across all affiliation categories, namely, *cell-cultured meat, cultured meat,* and *lab-grown meat* (Figure 2). While cell-cultured meat companies, research organizations, farmer advocacy groups, and animal welfare non-profits discussed all top 6 label terms, several affiliation groups completely omitted certain label terms. For example, traditional meat farmers predominately mentioned the terms *cell-cultured meat* and *cell-based meat* (Figure 2B), while omitting the label term *clean meat*. Similarly, government agencies did not mention *cultivated meat* or *cell-based meat* (Figure 2E), and *clean meat* was not mentioned in comments made by the “other” affiliation category (Figure 2G). Comments from unknown affiliations (Figure 2H) and government agencies (Figure 2D) scarcely mentioned any of the proposed labels.

### 3.3. Tyson foods, with interests in both cell-cultured and traditional meat, proposes three terms: *cultivated, cell-based, cultured*

Our third research question evaluated the comments submitted by Tyson Foods and how their suggested label terms compared to cell-cultured meat and traditional meat professionals. The Tyson Foods submission contained 1,669 words, 4 citations, and no supplemental information. Of the top label terms identified from the overall comments submitted to the ANPR (see Figure 2), only *cultured meat* and *cultivated meat* were mentioned by Tyson Foods. However, their submission mentioned a three-part conjoined term, “*cultivated/cell-based/cultured*” (mentioned 11 times), with the root term mentioned as being “along with the appropriate standard of identity or common or usual name.” When discussing these products, Tyson Foods most often referenced them as *cultured animal cells.* This suggests a work-around the existing limitations for cell-cultured meats with regards to the meat standard of identity regulation.

## 4. Discussion

The present study systematically evaluated comments submitted to a response ANPR that sought public input on cell-cultured meat labeling. Using our research hypotheses that economic interest and affiliation would affect submission extent and quantity, we found an effect opposite of what we expected. Further, the median length of submissions was highest for those without economic interest. This finding may be due to the nature of the organizations without economic ties submitting from the “other” and animal welfare non-profit affiliation categories, as they customarily voice their opinion on government policy. For example, within the affiliation category “other,” law students in the Harvard Animal Law and Policy Clinic and the Food Law and Policy Clinic submitted an 18-page response to the ANPR (33). They supported their sizeable comment by indicating the potential implications mandated labeling of these novel products has on First Amendment rights. Within the animal welfare non-profits, groups such as PETA submitted responses of length to express their outlook on the importance of these novel products for animal welfare. Three of the four submissions within this affiliation category responded to the prompted questions by the USDA-FSIS. While these organizations rely on voicing their opinions to guide policy, their lack of economic interest may also compel their extensive submissions as their relevance is not financially motivated, but ethically. Conversely, those with economic ties may not feel that it is necessary to support their argument in this manner as they are closely tied to the field, making their opinions inherently relevant.

While identified affiliation and economic interest were not predictors of submission quantity or length of submitted comments, percentages grouped by affiliation identified which types of organizations were most intrigued to submit. In terms of economic interest, we identified an overwhelming number of submissions from those with unknown ties. With this finding, we reject our first hypothesis that those with economic interest will have the highest number of submissions. It is also possible that these submissions chose to not disclose an economic interest. Only a small fraction of submissions were made by non-United States entities. There were 22 international submissions originating from Australia, Canada, Denmark, Germany, Israel, Netherlands, New Zealand, Saudi Arabia, Singapore, Spain, Uganda, and the United Kingdom. Of the international submissions, none had an identifiable organization. However, we found that 3 submissions from Canadian government agencies did not designate a country of origin; it is possible that other submissions did not declare their country of origin.

Interestingly, submissions made by individuals with unknown economic interest were much shorter as indicated by the lowest median word count of roughly a paragraph compared to those with and without economic interest of 1.5–3.5 standard pages. We infer that these submissions (i.e., with unknown ties) may represent the concerns and opinions of everyday consumers on how such products should be labeled. The differences in median word count could also be attributed to the lack of response to each of the 14 proposed rules that the FSIS provided with the ANPR. In general, comments that disclosed the submitting organization were much longer and provided comments on more of the 14 proposed rules – we believe that is reflective of organizational tie, regardless of economic impact.

Our findings regarding what types of affiliations submitted comments identified farmer advocacy groups to be the largest group of commenters with an identifiable organizational tie, submitting more than double the number of comments than the cell-cultured meat industry. It is apparent that these organizations likely voiced group opinions of traditional meat farmers as direct submissions from traditional meat farmers were minimal. Of the various farmer advocacy groups, it is known that the United States Cattlemen’s Association (USCA) is petitioning against the use of the term *meat* within labeling of meat alternative products, evidenced by their 2018 submitted petition to the FSIS to establish meat labeling requirements. This petition did not receive a response until 2021, in which the reply resulted in the release of the ANPR evaluated in this paper (34, 35). This suggests that the traditional meat industry has recognized cell-cultured meat commercialization as a significant impact to their livelihood and economy. Further, they advocate for transparent labeling that does not negatively impact profits of traditional meat products.

Our text analysis results identified several potential label terms for these novel food products, both with and without the root term ‘meat’. This forms a basis to further explore suitable labels. We quantified the percentages of label terms mentioned by each affiliation, which may provide evidence of how these affiliation groups differ in discussing these products. For example, the omission of *clean meat* by traditional meat farmers and the “other” affiliation category indicates that these groups do not identify it to be a potential label. We also found that all top 6 label terms were mentioned similarly when analyzed with and without the root term ‘meat’, which could indicate a labelling strategy including these hyphenated and preceding terms that would circumvent the potential restrictions of use of the term ‘meat’.

Our investigation of the most widely mentioned label terms allowed for visualization of the diversity in discussion of these novel products. Overall, *cultured meat* was mentioned in about 1/3 of comments, the highest percent of all top label terms identified (Figure 1A). However, when analyzing terms by affiliation category, differences in percentages of label mentions became apparent (Figure 2). Cell-cultured meat companies frequently mentioned the term *cultivated meat*, whereas traditional meat farmers frequently mentioned the terms *cell-cultured meat* and *cell-based meat*. This suggests that these two industries, thought to have directly opposing viewpoints, reference these products with different terminology. Our finding supports our second hypothesis regarding differences in predominant label terms mentioned in comments by these two affiliation categories. Further, the percent mentions by farmer advocacy groups revealed partial alignment with submissions by traditional meat farmers through similar percentages mentioning *cell-cultured meat*. The predominant labels *cultured meat* and *cell-cultured meat* by animal welfare non-profits may provide insight of how these products are being discussed by those who feel passionately about animal welfare. The scarcity of label mentions by government agencies and the unknown affiliate group makes it difficult to identify how these products are being discussed by these affiliations. However, from Supplementary Figure S2 it is apparent that these affiliation categories referenced these products primarily using the preceding term *cultured,* indicating use of a different root term or no root term at all.

We speculate that the hyphenated term *cell-among* the predominant label terms mentioned by traditional meat farmers, may indicate a preference for technology-oriented terminology. While labeling these products using the term *cell-has* been shown to increase consumer understanding, it may elicit a negative consumer response related to food technology neophobia, or perceived naturalness of these products (36). Further, the lack of the term *clean meat* in submissions made by traditional meat farmers may be due to the adverse effects that labeling cell-cultured meat as *clean meat* would have on the perception of traditional meat (inferring that traditional meat may be “unclean” or “dirty”). A recent study by Malerich & Bryant also scanned submissions to the ANPR to identify different labels for their consumer research study. They sought to identify consumer perception of the various label terms with regards to transparency, appeal, purchase intent, and perceived safety (16). While using the prefix ‘*cell-*’ increased consumer understanding of the product, however, appeal, purchase intent, and perceived safety for beef products was highest when presented with the term *cultivated meat* (16). As this term is the predominant label term mentioned by cell-cultured meat companies, we speculate that cell-cultured meat companies are proposing labeling of these products with more appealing terminology. Due to the limited analysis of the ANPR comments in Malerich & Bryant’s – in which they used an online tracker which only contained comments with an identifiable organization tie, and only analyzed the preceding label term, there are differences in the overall hierarchy of label terms identified in their and our study. Nonetheless, their findings confirm 5 of the top 6 labels we identified, with the except of the term *clean meat*, which we identified to be mentioned in 5% of comments.

We analyzed the submission by Tyson Foods separately due to their affiliation with both the traditional and cell-cultured meat industry. Their proposal of three potential terms *cultivated/cell-based/cultured* provides a term for each parameter of importance for a label: appeal (*cultivated*), understandability (*cell-based*), and neutrality (*cultured*) (37). Interestingly, each label term within the three-part conjoined term relates to the predominantly mentioned label terms by cultivated meat companies, traditional meat farmers, and overall submissions, respectively. Our findings suggest partial alignment with our hypothesis, namely, that Tyson Foods references these products using labels similar to those suggested by traditional meat farmers. However, we identified that the labels mentioned by Tyson were used by both traditional meat farmers and cell-cultured meat companies, which is opposite to what we initially hypothesized. Advantages and disadvantages for these three potential labels have been previously researched (38–41). While the term *cultivated meat* was found to provide greater consumer appeal, the term *cell-based meat* was more clearly understood (22). Label transparency was evaluated in a recent study by Hallman et al. (42). They also found that labels including the term “cell-” were more easily differentiated from traditional meat by a sample of 4,385 US consumers (42). Therefore, we speculate that major players within the cell-cultured meat industry may opt for a label that is both transparent and appealing to consumers.

Prior research has explored the use of different label terms with the public and measured consumer understanding, acceptance, perceived safety, and likelihood to purchase (16, 22, 43–45). Regardless of the lack of specified label terminology for such food products, a study in New Zealand suggested cell-cultured meat to be “rebranded as *clean meat,”* as recent studies demonstrated this label term was highest in positive consumer attitude and acceptance (45). While it is argued that this label leads to higher consumer acceptance, it is not recognized as being a neutral term and may create a negative bias on the traditional meat farming industry (22). The Good Food Institute (GFI) previously encouraged the use of the term *clean meat*, placing an emphasis on the importance of consumer appeal over neutrality and consumer understanding of the label (with the basis of all labels meeting a minimum standard of neutrality) (37). GFI provided one of the longest submissions to the ANPR with the most substantial supplemental materials (6 sections, 118 pages). While we were expecting submissions from academic researchers working in this area, we could not identify submissions from individuals working and publishing in the field. The submission from the Harvard Animal Law & Policy Clinic/Harvard Food Law & Policy Clinic on behalf of five academic researchers affiliated with this group, was the longest within the research organizations affiliation category and contained the largest number of scientific references. The most common term used throughout this submission was *cultivated meat.* In terms of relation to common everyday language, a paper evaluating tweets for mentions of cell-cultured meat indicates that *meat, cultured, cell, based, cultivated, clean,* and *cells* were among the top words used on Twitter about cell-cultured meat in the United States (46). This reinforces the focal points of a suitable label that were outlined previously and reiterates that the terms identified from social networks and the ANPR alike are commonly used among consumers and thus, provide understandable labeling strategies.

We analyzed all top preceding label terms (e.g., *cultured, cultivated, cell-cultured*) with and without the term meat. Previous research has shown that inclusion of the word *meat* does not play a significant role in consumer understanding (47). However, identifying these products as meat has implications for the standards of identity for meat and meat products as outlined in the introduction. This has become increasingly important with the commercialization of cell-cultured meat; the FSIS sought specific inputs on this issue in questions 7, 8, 10, and 11 (see Supplementary Table S1). As stated previously, not every submission provided comments on these specific questions. It is unclear why, but nonetheless, to use the term *meat* for these products, they either are recognized as meat under the current version of the CFR, or the CFR must be widened to encompass also cell-cultured meat. While cell-cultured meat has the potential to be recognized as a meat preparation process, to distinguish between cell-cultured meat and traditionally farmed meat, changes to 9 CFR317.2 to allow for cell-cultured meat labeling may in turn force the traditional meat industry to label their products as well. These labels may include terms such as “traditionally slaughtered” or “traditionally processed.” While the USDA-FSIS has not provided any follow-up announcements regarding the labeling of these products, it is currently stated on their website that “FSIS will ensure that cell-cultured products are labeled truthfully and consistent with coordinated FDA and FSIS labeling principles… FSIS does intend to publish new labeling regulations for such products” (48).

To further understand the use of the preceding terms by affiliation categories, we evaluated the percentage of comments for each preceding term of the top label terms (see Supplementary Figure S2). The majority of affiliation groups referenced all top preceding terms in at least 25% of submissions. While our analysis of label terms including the word *meat* revealed lower percentages of submissions referencing these terms, this may be attributed to the variety of root terms used (e.g., *protein, chicken, beef, cultured animal cells*). We also found that some submissions discussed using only the preceding term without a root term. Overall, proposed label term constructs varied in hyphenated, preceding, and root terms (see Supplementary Table S3). Those in support of cell-cultured meat commercialization often proposed consumer friendly label terms containing the root term ‘meat’ and used hyphenated and/or preceding terms such as *slaughter-free, ethical.* Those opposing commercialization often expressed that the term ‘meat’ should not be used to discuss these products. This may be due to the perception of these products as unnatural (49). Others felt that the term ‘meat’ was suitable, but in conjunction with preceding and/or hyphenated terms that may trigger disgust or neophobia within consumers (e.g., *synthetic, fake*) (36, 45). Further, some comments proposed novel terms (e.g., cegan, meatalin). Our findings exemplify the diversity in opinions on how these products should be labeled regarding all portions of label constructs (i.e., hyphenated, preceding, and root terms).

While a portion of submitters feel that these novel products should not be labeled with the root term ‘meat’, a similar case lies with plant-based meat and milk alternatives. The FDA has yet to establish appropriate labeling guidelines for these products. In January 2019, the National Milk Producers Federation (NMPF) submitted a comment to the FDA regarding the use of the word “milk” in plant-based products (32). The impact of labeling terminology on consumer choice and understanding of the nutritional value of plant-based milk products has reached significant concern at the level of the US government. In April 2021, the Dairy Pride Act was introduced in the Senate to enforce against misbranded milk alternatives (50). In 2022, the FDA has listed both of these topics as a part of the “Foods Program Guidance Under Development” (51). Draft recommendations for “the naming of plant-based foods that are marketed and sold as alternatives to milk” was released February 22, 2023, which ultimately allows for these products to be sold as “milk” and recommends a voluntary nutrient statement (52). No further recommendations have been made regarding the labeling of plant-based meat.

Due to the unique nature of our data sourcing, several limitations of our study exist. The low submission count from traditional meat farmers, in contrast to farmer non-profits and cell-cultured meat companies, impacts our comparison of label use rate between affiliate groups. Another limitation is our inability to identify organizational ties for the vast majority of submissions. Although it could be that these anonymous submissions were provided by everyday consumers, we can only assume these comments represent consumers’ attitudes toward labeling of cell-cultured meats. A limitation of our study design is that we quantified the percentage of label mentions rather than the direction of the statements relative to the labels (i.e., positive vs. neutral vs. negative sentiment). Sentiment analysis and other emotion analysis approaches could be performed to further understand negative and positive associations for each proposed label (53). This could consist of further identifying the support, or lack thereof, for each identified most common label term per submission.

Here, we set out to analyze the composition, extent, and disclosure of USDA ANPR submissions for the labeling of cell-cultured meat and poultry products. We identified several potential label terms for consideration. Future studies will evaluate each label term for consistency, transparency, and consumer acceptability. This may help explain *why* consumer appeal varies by label as evidenced by several studies (11, 29, 33). Once cell-cultured meat products are available for tasting, research on the interaction between label terms and perception will provide further insights into consumer acceptance of novel food products.

## Data availability statement

The original contributions presented in the study are included in the article/Supplementary material, further inquiries can be directed to the corresponding author.

## Author contributions

MF, HH, and JW conceived and designed the study. MF performed all text and statistical analysis with support from HH and JW. MF wrote the manuscript with input from HH and JW. HH and JW supervised the project. All authors contributed to the article and approved the submitted version.

## Funding

This work is supported by the USDA National Institute of Food and Agriculture and Hatch Appropriations under Project #PEN04699 and accession #1019351 to JW, project #PEN04792 and accession #7002577 to HH and a College of Agricultural Sciences SNIP grant to JW and HH.

## Conflict of interest

The authors declare that the research was conducted in the absence of any commercial or financial relationships that could be construed as a potential conflict of interest.

## Publisher’s note

All claims expressed in this article are solely those of the authors and do not necessarily represent those of their affiliated organizations, or those of the publisher, the editors and the reviewers. Any product that may be evaluated in this article, or claim that may be made by its manufacturer, is not guaranteed or endorsed by the publisher.

## Footnotes

^1^In this study, we will align with the USDA-FSIS referencing food products produced by cell-culture as “cell-cultured meat.”

^2^ https://www.regulations.gov/docket/FSIS-2020-0036/comments
