# Supplementary material for: Evaluation of public submissions to the USDA for labeling of cell-cultured meat in the United States
Source: Front Nutr. 2023 Sep 8;10:1197111. doi: 10.3389/fnut.2023.1197111 (PMC10514362; doi:10.3389/fnut.2023.1197111)
Supplement: Supplementary file 1 [file Data_Sheet_1.docx]

Supplementary Material

Evaluation of public submissions to the USDA for labeling of cell-cultured meat in the USA

Morgan Failla, Helene Hopfer, Josephine Wee*

*** Correspondence:** Josephine Wee: jmw970@psu.edu

# Supplementary Tables

***Table 1. 14 Issues for Comment Listed in the FSIS proposed rule.***

| **14 Issues for Comment** |
| --- |
| 1. Should the product name of a meat or poultry product comprised of or containing cultured animal cells differentiate the product from slaughtered meat or poultry by informing consumers the product was made using animal cell culture technology? If yes, what criteria should the agency consider or use to differentiate the products? If no, why not? |
| 2. What term(s), if any, should be in the product name of a food comprised of or containing cultured animal cells to convey the nature or source of the food to consumers? (*e.g.,* “cell cultured” or “cell cultivated.”)  a. How do these terms inform consumers of the nature or source of the product?  b. What are the benefits or costs to industry and consumers associated with these terms?  c. If meat or poultry products comprised of or containing cultured animal cells were to be labeled with the term “culture” or “cultured” in their product names or standards of identity (*e.g.,* “cell culture[d]”), would labeling differentiation be necessary to distinguish these products from other types of foods where the term “culture” or “cultured” is used (such as “cultured celery powder”)? |
| 3. If a meat or poultry product were comprised of both slaughtered meat or poultry and cultured animal cells, what unique labeling requirements, if any, should be required for such products? |
| 4. What term(s), if used in the product name of a food comprised of or containing cultured animal cells, would be potentially false or misleading to consumers? For each term, please provide your reasoning. |
| 5. What term(s), if used in the product name of a food comprised of or containing cultured animal cells, would potentially have a negative impact on industry or consumers? For each term, please provide your reasoning. |
| 6. Should names for slaughtered meat and poultry products established by common usage (*e.g.,* Pork Loin), statute, or regulation be included in the names or standards of identity of such products derived from cultured animal cells?  a. If so, is additional qualifying language necessary? What qualifying terms or phrases would be appropriate?  b. Do these names, with or without qualifying language, clearly distinguish foods comprised of or containing cultured animal cells from slaughtered products? |
| 7. Should terms that specify the form of meat or poultry products (such as “fillet”, “patty”, or “steak”) be allowed to be included in or to accompany the name or standard of identity of foods comprised of or containing cultured animal cells?  a. Under what circumstances should these terms be used?  b. What information would these terms convey to consumers? |
| 8. Should FSIS establish a regulatory standard of identity under its authorities in the FMIA and the PPIA (21 U.S.C. 607(c) and 457(b)) for foods comprised of or containing cultured animal cells?  a. If so, what would be the standard and how might compliance with the standard be verified?  b. If so, what would be the labeling terminology for products that do and do not meet a formal standard of identity? What would be the anticipated categories of use? For example, mechanically separated poultry that does not meet the standards of identity outlined in 9 CFR 381.173 may be diverted for production in broths and bases, as well as reaction flavors, *i.e.,* flavors produced by the heating of the protein source in the presence of a reducing sugar.  c. If so, what are the benefits and costs to industry if the standard of identity is established? Please provide quantitative and qualitative feedback in your response and explain the basis of any quantitative estimates.  d. If so, what are the consumer benefits and costs to the standard of identity recommended? |
| 9. What nutritional, organoleptic (*e.g.,* appearance, odor, taste), biological, chemical, or other characteristics, material to consumers' purchasing and consumption decisions, vary between slaughtered meat or poultry products and those comprised of or containing cultured animal cells? |
| 10. Should any of the definitions for “meat”, “meat byproduct”, or “meat food product” found in 9 CFR 301.2 be amended to specifically include or exclude foods comprised of or containing cultured animal cells? |
| 11. Should any of the definitions for “poultry product” or “poultry food product” found in 9 CFR 381.1 be amended to specifically include or exclude foods comprised of or containing cultured animal cells? |
| 12. Should FSIS-regulated broths, bases, and reaction flavors produced from cultured animal cells be required to declare the source material in the product name, ingredient sub-listing, or elsewhere on the label? |
| 13. Should the presence of cultured animal cells in further processed products regulated by FSIS, such as a lasagna made with cell cultured beef cells as an ingredient, be qualified on the product label? If so, how should this be qualified? |
| 14. What label claims are likely to appear on FSIS-regulated products comprised of or containing cultured animal cells? Should FSIS develop new regulations or guidance on such claims to ensure they are neither false nor misleading? |

***Table 2. Categories of Submitters, Explanation, and Economic Interest.*** *Each category of affiliation is presented with the explanation of submissions represented within the affiliate, and the percent of economic interest within the submissions by affiliation. Economic interest percent was calculated by quantifying the total submissions from each category that had economic interest and dividing by the total number of submissions within that affiliation category.*

| Affiliation | Explanation | Economic Interest |
| --- | --- | --- |
| Cell-cultured Meat Companies  *UPSIDE Foods*  *The Better Meat Co.*  *The Alliance for Meat, Poultry and Seafood Innovation*  *SVCMS*  *SuperMeat, the Essence of Meat*  *Orbillion Bio, Inc.*  *New Harvest*  *Matrix Meats*  *Future Meat Technologies*  *Fork & Goode*  *Finless Foods*  *BlueNalu, Inc.*  *Balletic, Inc.*  *Artemys Foods* | Included companies that produce cell-cultured meat. These include those that identify as cell-cultured meat investment companies. | 100% |
| Traditional Meat Farmers  *Exceldor Cooperative*  *Agri Beef* | Included companies that produce traditionally farmed meat. | 100% |
| Research Organizations  *True Health Initiative*  *The Vegetarian Resource Group*  *The Non-GMO Project*  *The Good Food Institute*  *International Food Information Council*  *FMI – The Food Industry Association*  *Center for Science in the Public Interest and Consumer Federation of America*  *Center for Foodborne Illness Research and Prevention, Ohio State University*  *Center for Food Safety & Food and Water Watch*  *Academy of Nutrition and Dietetics*  *A Greener World* | Included organizations that perform research relative to food science such as: agricultural environmental sustainability, human nutrition, food safety, and genetically modified foods. | 100% |
| Farmer Advocacy Groups  *US Beef Breeds Council*  *United Egg Producers*  *U.S. Cattlemen’s Association*  *Texas Farm Bureau*  *Texas Cattle Feeders Association*  *Texas & Southwestern Cattle Raisers Association*  *Tennessee Farm Bureau Federation*  *Southwest Meat Association*  *Pennsylvania Farm Bureau*  *Oklahoma Cattlemen’s Association*  *North Dakota Farmers Union*  *North American Meat Institute*  *New York Farm Bureau*  *NCC*  *National Turkey Federation*  *National Swine Registry & American Berkshire Association*  *National Pork Producers Council*  *National Milk Producers Federation*  *National Fisheries Institute*  *National Cattlemen’s Beef Association*  *National Association of State Departments of Agriculture (NASDA)*  *Missouri Farm Bureau*  *Kentucky Farm Bureau*  *Kansas Cattlemen’s Association*  *Iowa Cattlemen’s Association*  *Idaho Cattle Association*  *Georgia Farm Bureau*  *Further Processors Division of United Egg Association*  *Canadian Sheep Federation*  *Canadian Cattlemen’s Association*  *California Cattlemen’s Foundation*  *Arizona Farm Bureau*  *American Sheep Industry Association*  *American Grassfed Association*  *American Farm Bureau Federation*  *American Dairy Coalition*  *Alabama Farmers Federation* | Included organizations that advocate for traditional farming practices. | 100% |
| Federal, State, and Government Agencies  *United Senator Mike Rounds*  *Saskatchewan Ministry of Agriculture*  *North Dakota Dept of Agriculture*  *Montana Department of Livestock*  *Missouri Attorney General’s Office*  *Kentucky Livestock Coalition*  *Kentucky Department of Agriculture- Commissioner Ryan Quarles*  *Embassy of Canada to the US*  *Canadian Federation of Agriculture*  *California Department of Food and Agriculture; Animal Health & Food Safety Services; Meat, Poultry, and Egg Safety Branch*  *Arizona Department of Agriculture- Animal Services Division* | Included responses from government affiliated agencies and representatives. | 90.9% |
| Other  *Pet Food Institute*  *Olena Wellness*  *Mayan Beach Garden Hotel and Restaurant*  *Healthy Earth LLC*  *Harvard Animal Law & Policy Clinic and Harvard Food Law and Policy Clinic*  *Foley & Lardner LLP*  *Earthjustice*  *Earth Animal*  *Consumer Brands Association*  *BIOMILQ* | Recorded when affiliation did not fit into the categories presented. These included lawyers, plant-based food companies, other food companies, and pet food companies. | 33.3% |
| Animal Welfare Non-Profits  *PETA*  *Mercy for Animals*  *Compassion in World Farming*  *Animal Legal Defense Fund* | Included organizations that advocate for ethical farming practice. | 0% |
| No Affiliation | Included comments from those who did not identify with any organization. | UNKNOWN |

***Table 3. Examples of label constructs.***

| **Hyphenated terms** | **Preceding terms** | **Root terms** |
| --- | --- | --- |
| cell | cultured | meat |
| lab | cultivated | protein |
| animal | grown | *type of meat* (e.g., chicken, pork, beef) |
| meat | based | animal cells |
| man | artificial | *novel term* (e.g., cegan, meatalin, new age meat, pink slime) |
| cultured | fake | food product |
| authentic | made | *form of meat* (e.g., hamburger, nugget) |
| mutated | alternative | mass |
| cultivated | authentic | product |
| synthesized | animal | frankenfood |
| artificially | derived |  |
| genetically | cellular/cell(s) |  |
|  | nonmeat |  |
|  | synthetic |  |
|  | vegan |  |
|  | created |  |
|  | pure |  |
|  | clean |  |
|  | free |  |
|  | non-slaughtered |  |
|  | harvested |  |
|  | craft |  |
|  | engineered |  |
|  | raised |  |
|  | tech |  |
|  | manufactured |  |
|  | sustainable |  |
|  | brewed |  |
|  | imitation |  |
|  | eco |  |
|  | green |  |
|  | ethical |  |
|  | new |  |
|  | built |  |
|  | in vitro |  |
|  | humane |  |
|  | craft |  |
|  | faux |  |

***Table 4.*** *Presence of economic interests, citation of scientific evidence, supplemental information, and median word count*

| **Economic Interest** | **% of Total (n)** | **% Citing References**  **(n)** | **Mean Citation Count** | **Median Word Count (Range)** | **Comments with Supplemental Information** |
| --- | --- | --- | --- | --- | --- |
| Yes | 6.7 (77) | 32.5 (25) | 3.2 | 848  (202-5990) | 4 |
| No | 1.0 (12) | 50.0 (6) | 19.2 | 1766  (66-8731) | 2 |
| Unknown | 92.3 (1062) | 0.6 (7) | 0.1 | 68  (1-4609) | 6 |

# Supplementary Figures

***Figure 1. Workflow diagram of comment analysis.*** Data was sourced from the USDA-FSIS website [https://www.regulations.gov/docket/FSIS-2020-0036/comments], then cleaned-up using the described protocol.

***Figure 2. Percentages of submissions mentioning top label terms by affiliation without the root term ‘meat’.*** The percentages of submissions mentioning each of the top label terms, analyzed without the root term ‘meat’, was calculated for **(A)** cell-cultured meat companies (n=14), **(B)** traditional meat farmers (n=2), **(C)** research organizations (n=11), **(D)** farmer advocacy groups (n=37), **(E)** animal welfare non-profits (n=4), **(F)** government agencies (n=11), **(G)** other (n=10), and **(H)** unknown affiliations (n=1062).

**
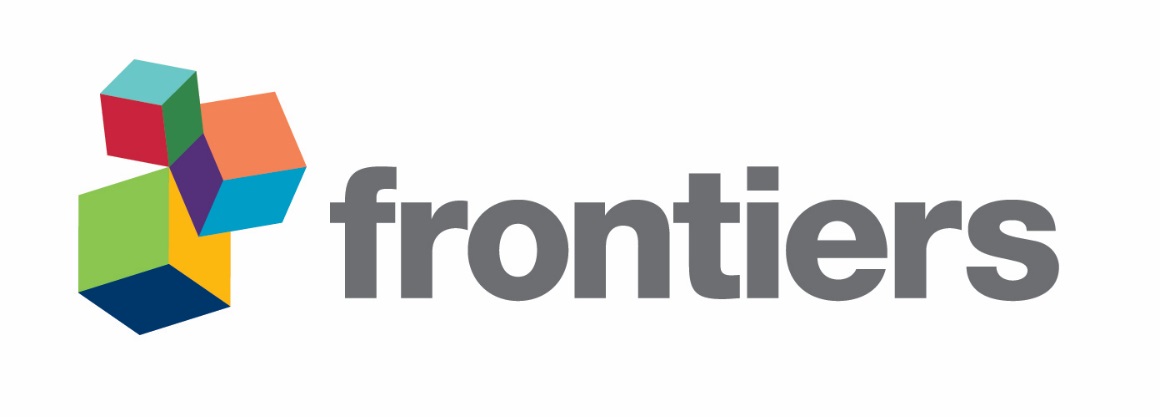
**
