# Supplementary figures and images for: Evaluation of public submissions to the USDA for labeling of cell-cultured meat in the United States
Source: Front Nutr. 2023 Sep 8;10:1197111. doi: 10.3389/fnut.2023.1197111 (PMC10514362; doi:10.3389/fnut.2023.1197111)

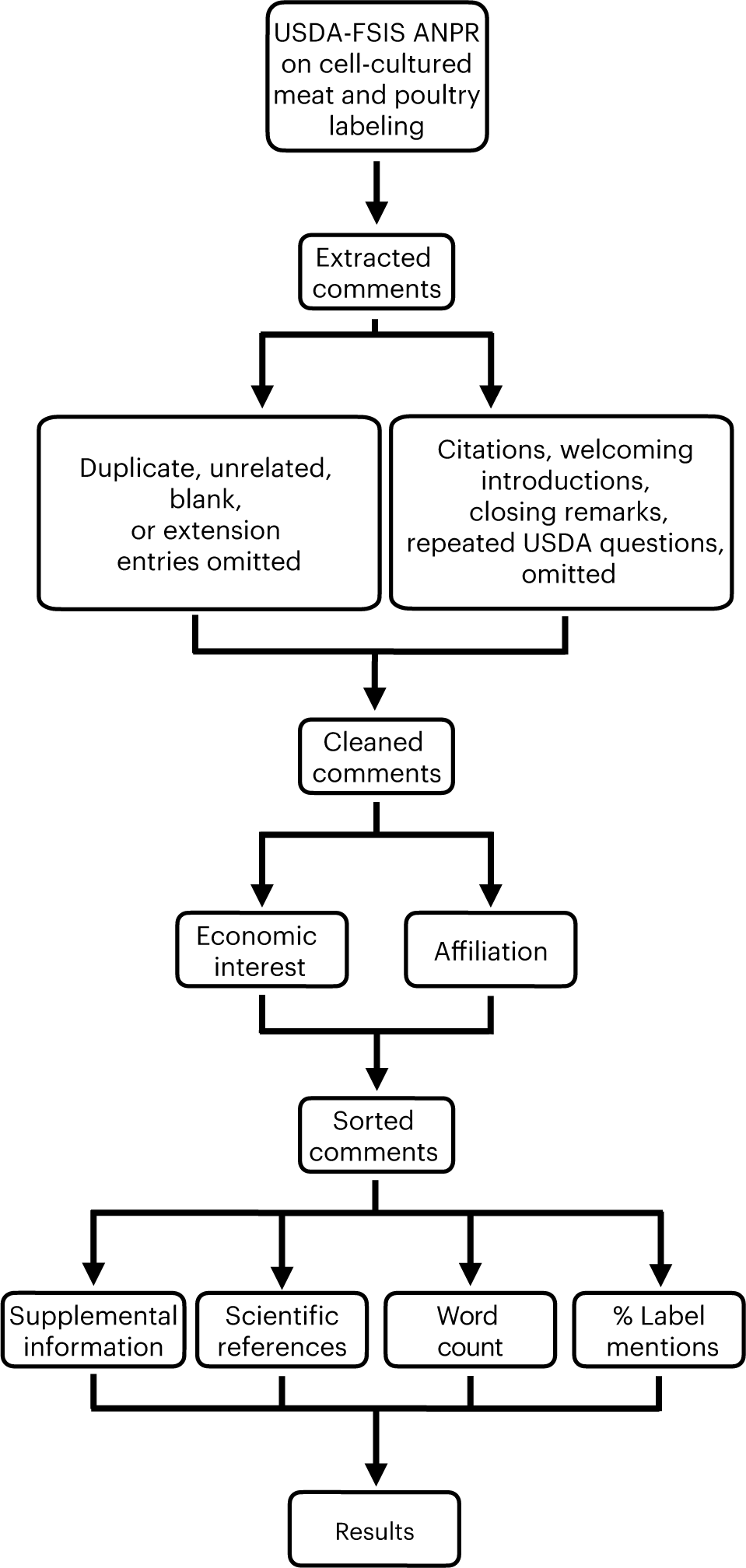

Supplement: Supplementary file 2 [file Image_1.TIF]

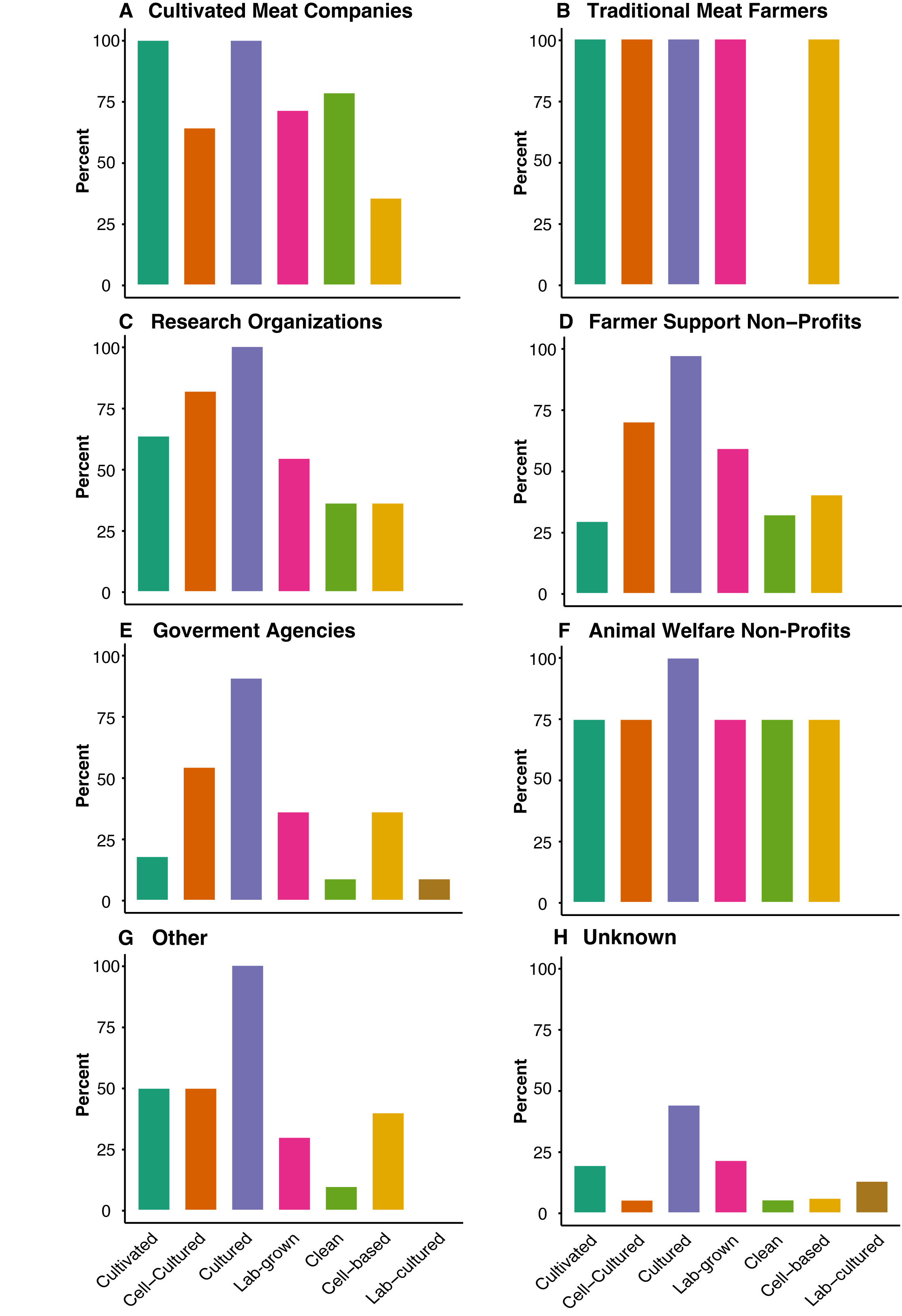

Supplement: Supplementary file 3 [file Image_2.TIF]
